# Intrasplenic Transplantation of Bioencapsulated Mesenchymal Stem Cells Improves the Recovery Rates of 90% Partial Hepatectomized Rats

**DOI:** 10.1155/2012/697094

**Published:** 2012-11-28

**Authors:** Zun Chang Liu, Thomas Ming Swi Chang

**Affiliations:** Departments of Physiology, Medicine, and Biomedical Engineering, Artificial Cells and Organs Research Center, Faculty of Medicine, McGill University, 3655 Promenade Sir William Osler, Room 1004, Montreal, QC, Canada H3G 1Y6

## Abstract

Mesenchymal stem cells (MSCs) derived from bone marrow can secrete cytokines and growth factors and can transdifferentiate into liver cells. We transplanted polymeric membrane bioencapsulated MSCs into the spleens of 90% partial hepatectomized rats. This resulted in 91.6% recovery rates. This is compared to a recovery rate of 21.4% in the 90% hepatectomized rats and 25% in the 90% hepatectomized rats receiving intrasplenic transplantation of free MSCs. After 14 days, the remnant livers in the bioencapsulated MSCs group are not significantly different in weight when compared to the sham control group. From day 1 to day 3 after surgery, in the bioencapsulated MSCs group, the plasma HGF and IL-6 were significantly higher than those in the free MSCs group and control group (*P* < 0.01); plasma TNF-**α** was significantly lower (*P* < 0.001). We concluded that the intrasplenic transplantation of bioencapsulated MSCs significantly increases the recovery rates of 90% hepatectomized rats. It is likely that the initial effect is from proliver regeneration factors followed later by the transdifferentiated hepatocyte-like cells. However, histopathological analysis and hepatocyte proliferation study will be needed to better understand the regenerative mechanisms of this result. This study has implications in improving the survival and recovery of patients with very severe liver failure due to hepatitis, trauma, or extensive surgical resection.

## 1. Introduction

There are mainly two types of stem cells in the bone marrow, hematopoietic stem cells (HSCs) and mesenchymal stem cells (MSCs). Bone marrow MSCs secrete several factors and cytokines including interleukin 6 (IL-6) and hepatic growth factors that contribute to liver regeneration [1, 2]. Therefore, MSCs might play a role in increasing the regeneration of the liver. There are a number of ways to investigate the use of MSCs and other cells [3]. Each of these has its own advantages depending on the specific area of use [3]. For our specific study on liver regeneration, we are investigating the principle of polymeric membrane microcapsules [4–8] to bioencapsulate bone marrow MSCs, for the following reasons.

1. Cells are retained inside polymeric membrane artificial cells, thus protected from immune rejection-immunoisolation [4–9].
2. There has been much study on cell bioencapsulation for cell therapy using islets, hepatocytes, genetic engineered cells, and other cells with promising results [5–8]. However, more research is needed towards long-term biocompatibility to allow implanted bioencapsulated cells to work for more than 1 year after implantation. We, therefore, look for the use of bioencapsulated stem cells for liver regeneration in acute liver failure. In this case, implanted bioencapsulated MSCs only need to function for a relatively short period of time (weeks in rats and months in human) to enhance liver regeneration into a functioning liver.
3. Bioencapsulated MSCs are retained and immunoisolated inside the polymeric membrane microcapsules. This also avoids the problem of dispersal of implanted free MSCs to avoid the rare but potential tumorigenicity of MSCs.

We started by using polymeric microcapsules [4–6] to bioencapsulate bone marrow nucleated cells for intraperitoneal injection into 90% hepatectomized rats [9]. This resulted in an increase in the recovery rates of the 90% hepatectomized rats and regeneration of the remnant liver [9]. We hypothesized that this was due to two factors. First, there was the immediate mediating effect of hepatocyte growth factor (HGF), secreted from the bioencapsulated MSCs after transplantation, such growth factors stimulated and accelerated remnant liver regeneration [5, 6, 9]. This was followed by the transdifferentiation of MSCs into hepatocytes to maintain some liver functions until the liver regenerates sufficiently [5, 6, 9]. However, intraperitoneally injected bioencapsulated stem cells are dispersed throughout the peritoneal cavity and not localized, and this may irritate the peritoneal lining to cause peritonitis. Spleen is a possible site for cell transplantation in the treatment of various liver diseases [10–13], especially when the liver is not suitable as a transplantation site. However, intrasplenically transplanted cells could migrate to the liver via the portal vein and cause embolism in the intrahepatic portal vein system [13]; therefore, in the present study, we bioencapsulated MSCs using alginate polylysine alginate (APA) membrane and transplanted them into the spleens of 90% hepatectomized rats. This way, the bioencapsulated MSCs would stay in the spleen after transplantation. Unlike free MSCs, the bioencapsulated MSCs are retained at all time and do not become dispersed to cause adverse effects. For 14 days after transplantation, we followed the percentage of recovery in the 90% PH rats, the weight of the remnant livers, the blood levels of cytokines, and growth factors.

## 2. Results

### 2.1. The Percentage of Recovery in 90% Hepatectomized Rats

 The experiment endpoint is 14 days. The clinical endpoints are when the clinical conditions of the rats have reached the terminal stage that include ascites, labored respiration, and hunched posture. If these clinical endpoints appeared, the rats were euthanized. Those that have not reached the clinical endpoint are counted as “recovered.” 

**Figure 1:**
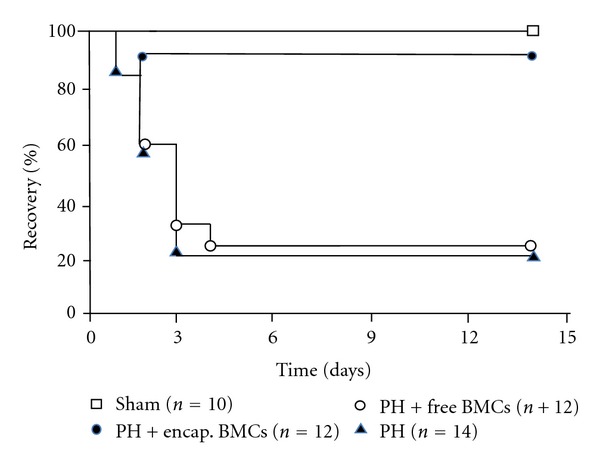
The percentage of recovery of rats.

The percentage of recovery 2 weeks after transplantation shows the following results. Implantation of bioencapsulated MSCs into the spleens significantly increased the recovery of the 90% hepatectomized group compared to the untreated group (*P* < 0.05, Figure 1). However, implantation of free MSCs into the spleens of the 90% hepatectomized group did not result in any significant changes when compared to the untreated group (*P* > 0.05, Figure 1).

The experiment endpoint is 14 days. The clinical endpoints are when the clinical conditions of the rats have reached the terminal stage. Those that have not reached the clinical endpoint are counted as “recovered” as shown in Figure 1. Lines starting from top down: (1) Sham control; (2) 90% hepatectomized (HP) + bioencapsulated MSCs; (3) PH + free MSCs; (4) PH. The percentage of recovery rates is significantly higher in the bioencapsulated MSCs group when compared with the free MSCs group or the PH control group (*P* < 0.05).

### 2.2. Liver Wet Weight in Different Groups

**Figure 2:**
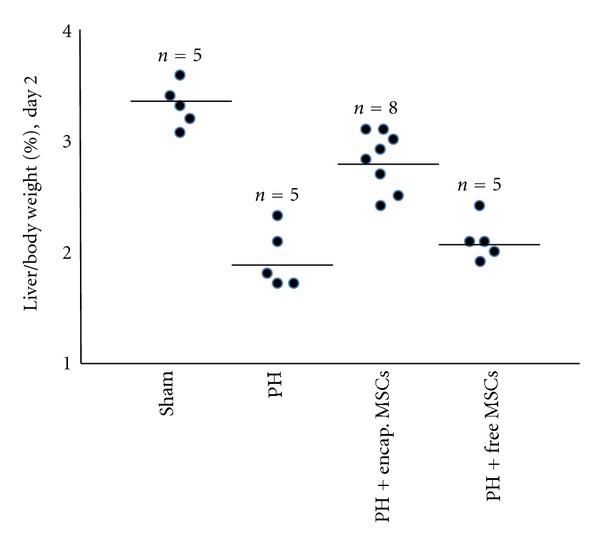
Remnant liver wet weight on day 2.

**Figure 3:**
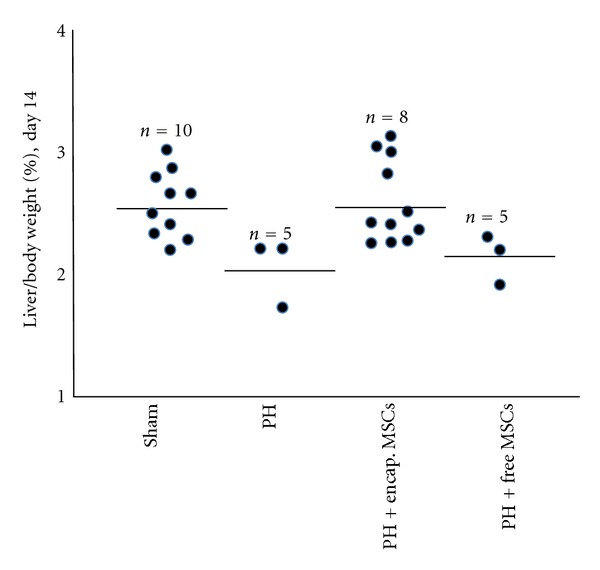
Remnant liver wet weight on day 14.

 On day 2 after transplantation, there was no significant difference between the free MSCs group and 90% PH control group in the liver to body weight ratio (*P* > 0.05, Figure 2). On the other hand, as early as day 2 after transplantation, the liver to body weight ratio in the 90% hepatectomized group receiving bioencapsulated MSCs was significantly higher than that in the untreated 90% hepatectomized group and free MSCs group (*P* < 0.001, Figure 2). However, the liver to body weight ratio was still significantly below that of the sham control group on day 2. It was after 14 days that the liver to body weight ratio of the bioencapsulated MSCs group reached that of the sham control group (Figure 3). This shows that in the bioencapsulated MSCs transplantation group, after 2 days of transplantation, the liver mass recovered faster than the PH control and free MSCs transplantation, but still not restored to the full original size at this time until day 14. This significant increase in liver mass may have helped to maintain sufficient liver function for the rats to survive until the liver has regenerated. 

On day 2 in the bioencapsulated MSC group, the remnant liver to body weight ratio was significantly higher than those in the PH control and free MSCs group (*P* < 0.001), but was still lower than that in the sham group (*P* < 0.01).

On day 14 after 90% PH, the remnant liver wet weight to body weight ratio in those animals in the bioencapsulated MSCs group that have recovered was not significantly different than the sham control group (*P* > 0.05). 

### 2.3. Growth Factors and Cytokines in Plasma

**Figure 4:**
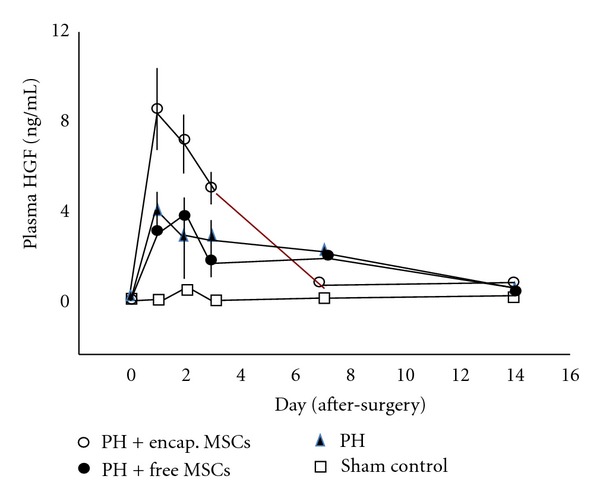
HGF in plasma.

**Figure 5:**
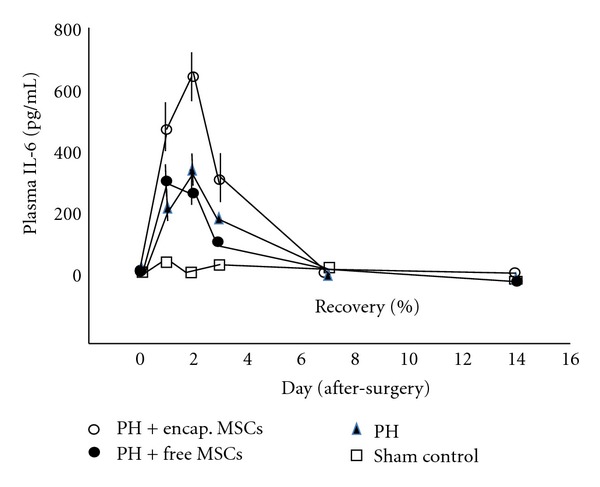
IL-6 in plasma.

**Figure 6:**
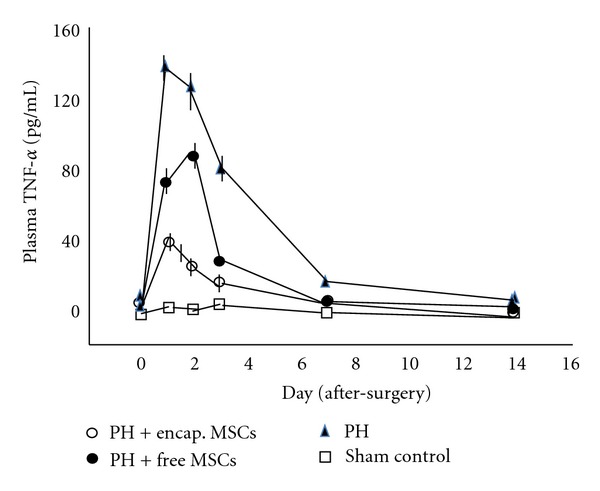
TNF-*α* in plasma.

In the bioencapsulated MSCs transplantation group, the HGF and IL-6 levels were higher than in the 90% PH control and free MSCs group from day 1 to day 3 after transplantation (*P* < 0.01; Figures 4 and 5), whereas the TNF-*α* was higher in the 90% PH control and free MSCs group from day 1 to day 7 after transplantation than in the bioencapsulated MSCs group (*P* < 0.01, Figure 6).

From day 1 to day 3 after surgery, in the bioencapsulated MSCs group, the plasma HGF was significantly higher than in the free MSCs group and control group (*P* < 0.01). 

During the first three days after surgery, IL-6 was significantly higher in the bioencapsulated MSCs group than in the 90% PH control and free MSCs group (*P* < 0.01).

Plasma TNF-*α* in the 90% PH control was significantly higher than that in the bioencapsulated MSCs group and the free MSCs group in the first 3 days after surgery (*P* < 0.001). In the free MSCs group, the TNF-*α* was significantly higher than in the bioencapsulated MSCs group (*P* < 0.01). 

## 3. Discussion

Intrasplenic transplantation of bioencapsulated MSCs significantly increases the percentage of recovery in 90% PH rats, whereas this effect was not observed in the free MSCs transplantation group.

The mechanism of liver failure after excessive hepatectomy is not clear. It may involve loss of functional parenchyma cells, acute inflammatory reaction of remaining liver, and the microcirculation disturbance. After massive hepatectomy, fatal hepatic failure is characterized by increased apoptosis and diminished liver regeneration; so there is insufficient liver capacity to satisfy metabolic demand of the body and maintain homeostasis [14]. After PH, the flow injury to sinusoidal endothelial cells activates Kupffer cells which release inflammatory cytokines like TNF-*α* and TGF-*β*1. These further induced necrosis and apoptosis of the remnant liver [15–17]. In our study in the first several hours after PH, TNF-*α* takes part in the priming step of liver regeneration, this priming step induces the transition of cells from G0 to G1 [18, 19]. Thereafter, the sustained elevated TNF-*α* induces hepatocytes apoptosis of the remnant liver [20]. In the present study, there was a significant increase of blood TNF-*α* 24 hours after 90% PH. The high level of TNF-*α* may have induced further hepatocytes apoptosis at this stage. TNF-*α* was significantly lower in the bioencapsulated MSCs after 24 hours transplantation when compared to the PH control and free MSCs transplantation groups. 

HGF and IL-6 in the bioencapsulated MSCs group were higher than those in the PH control. It is likely that this is due to the production of HGF and IL-6 from the bioencapsulated MSCs. Previous in vitro study shows that bioencapsulated MSCs produced IL-6 and HGF [5, 6, 9]. HGF and IL-6 are important growth factor and cytokine of proliver regeneration after liver failure [21–24]. Furthermore, IL-6 stimulates the reentry of quiescent cells into the cell cycle within the first 2 to 4 hours after PH [18]. 

Our earlier intraperitoneal implantation study shows that some of the bioencapsulated MSCs on day 14 after transplantation showed transdifferentiation into hepatocyte-like cells [9]. However, we did not find any transdifferenitation on day 2 after transplantation [9]. Since most of the animals that died were within the first 3 days of 90% hepatectomy, it would appear that it was the factors such as HGF and IL-6 secreted from the bioencapsulated MSCs that played the initial roles in enhancing liver regeneration and preventing the necrosis and apoptosis of remnant liver. Later, the bioencapsulated MSCs in the spleen are transdifferentiated into hepatocyte like cells. This way, the spleen may have functioned as an ectopic liver support with some liver functions to maintain the life of the HP rats until the liver has regenerated. 

 There are a number of reasons for intrasplenic transplantation of bioencapsulated MSCs. Firstly, after 90% PH, the remnant liver is too small to be transplanted with large amount of bioencapsulated MSCs. Secondly, previous studies showed the MSCs transplanted via systemic circulation might reside in liver and differentiate to stellate cells and myofibroblasts, both of which can contribute to fibrosis and cirrhosis of liver [25, 26]. In our study, we confined the MSCs in the spleen by the bioencapsulation of the MSCs. Thirdly, the growth factors and cytokines produced from the intrasplenic transplanted MSCs can drain directly to the remnant liver, keeping the factors in high concentration in the portal system and in the remnant liver. Fourthly, bioencapsulated MSCs in the spleen can no longer leave the spleen to cause embolism. This would solve the problem of embolism in the intrasplenic transplantation of free MSCs previously reported [27, 28]. This way, the MSCs are retained inside and do not disperse throughout the body in the unlikely possibility of mutation of MSCs to form carcinogenic cells. Our results showed that unlike free MSCs, bioencapsulated MSCs stayed inside the spleen when followed up to 14 days. 

Polymeric microcapsules for bioencapsulation of different types of cells have been extensively investigated with promising results [5–8]. However, they can only function for up to 12 months after implantation because of long-term biocompatibility problems [5–9]. However, in the case of the present study where liver regeneration can take place within a comparatively short time, the bioencapsulated cells only need to function for this duration of time without problems related to long-term biocompatibility.

 In summary, in the 90% hepatectomized rat model, intrasplenic transplantation of bioencapsulated MSCs enhances liver regeneration and improves the recovery rates of the animals. This is likely because of two reasons. At the beginning, the bioencapsulated MSCs secrete hepatic growth factor and cytokines that drain into the remnant liver via the portal vein resulting in enhanced liver regeneration. Later, the bioencapsulated MSCs in the spleen are transdifferentiated into hepatocyte like cells. This way, the spleen may have functioned as an ectopic liver support. Further research on the basic aspect of liver regeneration in this approach needs to be carried out. For instance, detailed histopathological studies should be carried out to study this in more detail. In addition, hepatocyte proliferation data should be collected using either BrdU or Ki-67 (MIB-5). If MSCs improve regeneration, this would be important to show on a daily basis for the first few days after PH. In addition, it should be pointed out that this is a rat model, and hepatic regeneration in rats is much faster than that in human. Thus, the result of this study cannot be directly applied to human until further research is carried out in other animal models.

## 4. Materials and Methods

### 4.1. Animals

Male Wistar rats, 200–225 g, purchased from Charles River (St-Constant, Canada) were donors for bone marrow cells. Syngeneic male Wistar rats were used as the recipients. 

### 4.2. Bone Marrow Stem Cells Isolation and MSCs Expansion

Wistar rats were anaesthetized with sodium pentobarbital and both femurs were isolated. Serum-free L-DMEM (low glucose DMEM, GIBCO, BRL) was used to flush out bone marrow cells from the femurs using a 5 mL syringe with a 22-gauge needle. Bone marrow mononuclear cells were isolated with Percoll gradient density (Sigma, MO, USA 1.073 g/mL) centrifugation. Briefly, cell suspension was layered over an equal volume of Percoll solution, centrifuged at 2000 rpm/min for 20 min. We then recover the white layer which was the mononuclear cells right below the plasma layer. Cells were resuspended in expansion medium (DMEM low glucose, 10% FBS, 2 mM L-glutamine, HEPES, 100 U/mL penicillin and 100 *μ*g/mL streptomycin, Amphotericin B 2.5 *μ*g/mL, 10 ng/mL epidermal growth factor (EGF), 10 ng/mL bFGF). They were seeded in 10 cm culture dishes at a density of 5 × 10^4^ cells/cm^2^, incubated in 95% air, 5% CO_2_ at 37°C, with fresh medium change every 3-4 days. The adherent cells were allowed to reach 80% confluence, then passage [29, 30]. Usually after 3 passages, the cells are purified as spindle-shaped MSCs and could be harvested for further experiment use.

### 4.3. Bioencapsulation of MSCs

Alginate polylysine alginate (APA) microencapsulation method was used to encapsulate the MSCs as described previously [6, 9, 31]. Briefly, MSCs 2 × 10^8^ were suspended in 15 mL 1.5% sodium alginate solution (Inotech, Rockville, USA). The cell suspension was extruded through droplet generator (NISCO Encapsulator, NISCO Engineering AG, Switzerland). The beads formed were allowed to fall into a PYREX dish containing 100 mM CaCl_2_. After the beads were allowed to gel in the calcium solution for 5 min, the beads were immersed in 50 mg% poly-L-lysine solution for 15 min, washed with buffered saline (0.85% NaCl, 10 mM HEPES, 20 mM D-fructose, PH 7.4), and then immersed into 0.2% sodium alginate for 10 min. Finally, the beads were placed in 50 mM sodium citrate for 20–30 min to dissolve the inner alginate gel and to form an APA membrane. The final microcapsules containing MSCs were incubated in L-DMEM without supplements, serum-free, in 5% CO_2_, 37°C incubator for 4 hours prior to transplantation.

### 4.4. Ninety Percent PH and Transplantation Protocols

The 90% PH was performed according to the standard procedure [32]. For the sham operation, we only carried out abdomen incision and cutting of the suspending ligament of the liver and then closed the incision. Immediately after 90% PH, MSCs bioencapsulated MSCs were injected intrasplenically using an 18-gauge needle and 23 G needle for free MSCs injection. Injections were made through 3-4 sites in the spleen. Absorbable hemostat (Surgicel NU-KNIT, Ethicon Inc., NJ, USA) was used to prevent bleeding after injection.

We performed two animal experiments, in the first experiment, 48 animals were randomly divided into 4 groups: sham control (*n* = 10); 90% PH control (*n* = 14); 90% PH transplanted with 3 × 10^7^ bioencapsulated MSCs (*n* = 12); 90% PH transplanted with 3 × 10^7^ free MSCs (*n* = 12). Blood samples were taken from these rats at different time points. The experiment endpoint for this group is 14 days. The experiment endpoint is 14 days. The clinical endpoints are when the rats have reached the terminal stage that include ascites, labored respiration, and hunched posture. If these clinical endpoints appeared, the rats were euthanized. Those that have not reached the clinical endpoint are counted as “recovered.” 

 In the second animal experiment, 31 rats were divided into the following groups and received the respective intervention, sham control (*n* = 5); 90% PH control (*n* = 9); 90% PH transplanted with 3 × 10^7^ bioencapsulated MSCs (*n* = 8); 90% PH transplanted with 3 × 10^7^ free MSCs (*n* = 9). 

 McGill University's institutional animal ethic committee does not allow survival study to death. Thus, the experiment endpoint for this group is 2 weeks. If signs of clinical endpoints appear, such as terminal stress like ascites, labored respiration, hunched posture, then the rats were euthanized. At day 14 after PH, all rats alive to the experiment endpoint were sacrificed, the liver and spleen were resected for analysis.

### 4.5. Blood Cytokines Assay

Blood samples were taken from recipient rats before and at different time points after PH. Plasma samples were stored in −76°C freezer until tested. The HGF, TNF-*α*, and IL-6 in plasma was measured using enzyme-linked immunosorbent assay (ELISA). HGF ELISA kit was purchased from Institute of Immunology, Tokyo, Japan. TNF-a and IL-6 kits were purchased from R&D Systems, Minneapolis, MN, USA. Analysis procedures were following the manufacturers' instructions.

### 4.6. Statistical Analysis

Data were presented as mean ± SD. For multiple comparisons of more than two groups, we performed one-way or two-way analysis of variance (ANOVA). If the ANOVA was significant, we performed the Newman-Keuls procedure as a post hoc test. Kaplan-Meier curve was used to analyze the percentage of recovery rats. *χ* ^2^ (chi-square) method was used for comparing the percentage of recovery in each group. A *P* value of less than 0.05 was considered significant.
